# Exosomes: A Novel Strategy for Treatment and Prevention of Diseases

**DOI:** 10.3389/fphar.2017.00300

**Published:** 2017-06-13

**Authors:** Jiaqi Wang, Xiaoyan Sun, Jiayu Zhao, Yang Yang, Xueting Cai, Jianguang Xu, Peng Cao

**Affiliations:** ^1^Hospital of Integrated Traditional Chinese and Western Medicine, Nanjing University of Chinese MedicineNanjing, China; ^2^Laboratory of Cellular and Molecular Biology, Jiangsu Province Academy of Traditional Chinese Medicine and Jiangsu Branch of China Academy of Chinese Medical SciencesNanjing, China; ^3^Department of Endoscopy, Quzhou People’s HospitalQuzhou, China

**Keywords:** vesicles, exosomes, pathogenic microorganism, cancer, diagnosis index, drug delivery

## Abstract

An “exosome” is a nanoscale membrane vesicle derived from cell endocytosis that functions as an important intercellular communication mediator regulating the exchange of proteins and genetic materials between donor and surrounding cells. Exosomes secreted by normal and cancer cells participate in tumor initiation, progression, invasion, and metastasis. Furthermore, immune cells and cancer cells exert a two-way bidirectional regulatory effect on tumor immunity by exchanging exosomes. Current studies on exosomes have further expanded their known functions in physiological and pathological processes. The purpose of this review is to describe their discovery and biological functions in the context of their enormous potential in the clinical diagnosis, prevention, and treatment of cancer as well as bacterial and viral infectious diseases.

## Introduction

The concept of “exosomes” was originally referring to membrane vesicles obtained from biological fluids following studies that reported the secretion of 50 nm-sized vesicles from reticulocytes, which were associated with the transferrin receptor during the maturation process (Harding and Stahl, 1983; Pan and Johnstone, 1983). Later, Rose Johnstone used the term exosome to refer to extracellular vesicles (EVs), a concept which has garnered increasing awareness and the term is in current use (Trams et al., 1981). In 2013, Randy Schekman, James Rothman, and Thomas C. Südhof were awarded the Nobel Prize in Physiology or Medicine for their discovery of the regulatory mechanism of the main intracellular transport system of the cell, the vesicle transport system. With further study and increasing awareness of the role and function of exosomes, what was originally disregarded as a simple vesicle has become a hot research topic that has attracted the attention of researchers worldwide, who have discovered that exosomes play important roles in many physiological and pathological processes.

Various types of mammalian cells release EVs into surrounding tissues or cells for intracellular communication. Being generally recognized as having a secretory vesicle structure, EVs are currently divided into two main categories: exosomes derived from the endocytic pathway or microvesicles derived from cell membrane shedding, which includes ectosomes and microparticles (Harding and Stahl, 1983). In addition to differences in origin, exosomes from these categories have certain differences in their molecular characteristics and mode of extraction. Exosomes refer to the membrane vesicle structure derived from cell endosomes, with a molecular size of 30–100 nm (Webber et al., 2010). In the context of this review article, we consider exosomes in the context of major EVs. Originally, exosomes referred to vesicles with a molecular size of 40–100 nm that are secreted by reticulocyte cells during differentiation; however, it was later discovered that B lymphocytes and dendritic cells (DCs) also secrete exosomes through a similar process (Raposo et al., 1996; Zitvogel et al., 1998). In addition, it was demonstrated that hematopoietic and non-hematopoietic cells, such as cytotoxic T cells, platelets, neuronal cells, and mast cells, release exosomes by integrating intracellular multivesicular endosomes (MVEs) with the cell membrane (Clotilde et al., 2009; Simons and Raposo, 2009). Accordingly, exosomes are nanosized membrane vesicles released by many kinds of cells during the fusion of intracellular MVEs and the cell membrane (Harding et al., 1984; Pan et al., 1985).

### Classification of EVs

Extracellular vesicles are small spherical bio-membrane structures, which include microparticles and exosomes (Gould and Raposo, 2013; Raposo and Stoorvogel, 2013), secreted by cells, such as endothelial cells, cancer cells and pathogens (e.g., bacteria and viruses), (Kawamoto et al., 2012; Yamamoto et al., 2015). Based on differences in biosynthesis and size, EVs are divided into three subgroups: membrane-shedding EVs (microparticles), multivesicular body-derived EVs (exosomes), and apoptosis-derived EVs (apoptotic bodies). Microparticles (also known as microvesicles) are medium-sized vesicles (50–3,000 nm) and apoptotic bodies are larger vesicles (800–5,000 nm), whereas exosomes are comparatively smaller-sized vesicles (40–100 nm) (Yamamoto S. et al., 2016).

In recent years, exosomes have received significant attention from researchers because they transport functional molecules, such as messenger RNA (mRNA), microRNA (miRNA), and protein, into target tissues or cells. These bioactive molecules are considerably stable and can modulate cell behaviors in recipient cells. There is increasing evidence supporting the hypothesis that exosomes secreted by various cells play roles in many physiological and pathological processes, especially in cancer. In this review, we briefly summarize the most recent data and findings concerning the function of exosomes and discuss their potential role in diagnosis and therapeutic regimens of human diseases.

## The Origin and Characteristics of Exosomes

**FIGURE 1:**
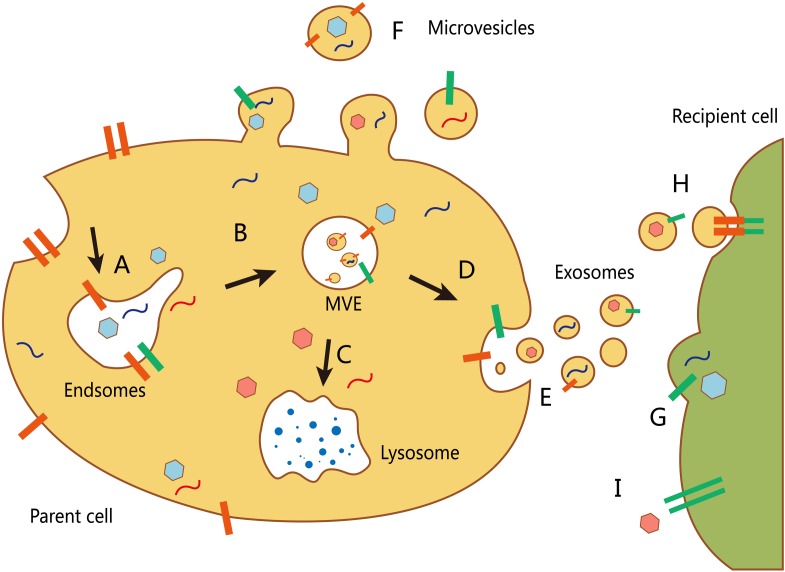
**Biogenesis and transport of different extracellular vesicles (EVs). (A)** The formation of an early endosome. **(B)** The development of an early exosome into a late endosome (multivesicular body, MVB). The inward budding allows packing of cytosolic contents in the intraluminal vesicles. **(C)** The fusion of mature MVBs and lysosome to degrade the vesicle cargo. **(D)** MVBs can also fuse with the plasma membrane to release their intraluminal vesicles (as exosomes). **(E)** Secretion of exosomes into the extracellular environment. **(F)** Other microvesicles can be secreted through direct budding from the plasma membrane of the host cell. There are at least three mechanisms by which EVs interact with recipient cells: **(G)** direct fusion with the plasma membrane of the recipient cell; **(H)** receptor-mediated endocytosis following receptor–ligand interaction between EVs and the recipient cell; and **(I)** signaling via direct interactions of the receptor and its ligand on the recipient cell surface.

The inward depression of the cell membrane may form early endosomes, whereas a small part of an early endosome can react with the cell membrane and be released, which forms a microvesicle. Early exosomes then develop into late endosomes, namely MVEs, that either combine with a lysosome and digest its contents or be released as an exosome through exocytosis (Booth et al., 2006) (**Figure 1**). The main difficulty faced in exosome research is improvement of available EV extraction and analysis methods. Currently, differential velocity centrifugation methods are used to extract exosomes from the supernatants of fetal bovine serum cultured cells in the absence of cattle-derived EVs. Exosomes and other EVs are spherical and have a lower buoyant density than that of other non-membrane fragments (Van et al., 2003). Further, different suspension rates are used to distinguish EVs with different molecule sizes (Wubbolts et al., 2003).

Exosomes have an extracellular membrane vesicle structure composed of a phospholipid bilayer membrane. Their surface membrane contains various proteins, such as CD63, CD81, CD82, CD53, and CD37 (Hemler, 2013), which can be used as biomarkers of cell membrane and early exosome origins (Escola et al., 1998). Exosomes from various sources contain several types of exosome-associated proteins, such as Rab GTP enzyme, soluble N-ethylmaleimide-sensitive factor attachment protein receptors (SNAREs), membrane-associated proteins, and lipid raft structural proteins. Compared to the cell membrane, exosomes are rich in cholesterol and sphingomyelin (Brouwers et al., 2013), but also contain some proteins associated with lipid rafts including glycosylation phosphatidylinositol immobilized protein and lipid raft structural protein (Wubbolts et al., 2003). Exosomes secreted from the donor cell can transport many biological components, such as functional mRNAs, miRNAs, DNA fragments, lipids, and proteins, into recipient cells (Ratajczak et al., 2006; Valadi et al., 2007). Exosomes enable the contents of the vesicle to be stably maintained for long-distance transport. Therefore, transport via exosomes is an effective way to influence not only surrounding cells but more distant target cells and even systemic responses. Initial studies investigated exosome release from reticulocytes as a mechanism of removing waste molecules from cells (Harding et al., 2013), whereas other studies proposed that there was functional communication between EVs and cells citing as an example that proteasomes can promote sperm motility (Stegmayr and Ronquist, 1982). In recent years, an increasing number of physiological functions of exosomes have been reported and their crucial roles in intercellular communication have been gradually realized.

Following their release into the extracellular environment, exosomes integrate with target cells through three mechanisms. Recipient cells that are near donor cells integrate and absorb exosomes through endocytosis, while recipient cells that are slightly distant absorb them through the paracrine pathway. Other more-distant recipient cells absorb exosomes through circulation and the endocrine pathway. The endocytotic process of recipient cells is associated with regulation of a surface-specific molecule or a four-transmembrane domain protein receptor from recipient cells, which indicates the uptake of exosomes is not completely random.

It is currently believed that exosomes mediate information exchange between cells through four ways: (1) Exosomes are used as a signal complex directly stimulating recipient cells through binding to a cell surface ligand; (2) Exosomes transfer receptors between cells; (3) Exosomes deliver functional proteins or infectious particles to recipient cells; and (4) Exosomes transfer genetic information to recipient cells through mRNAs, microRNAs, or transcription factors (Camussi et al., 2010). Once exosomes are absorbed by the recipient cell, stored lipids, proteins, mRNAs, miRNAs, and other molecules can then affect the function and cellular phenotype of the recipient cell by regulating signal cascade pathways, key enzyme reactions, cellular homeostasis, or other mechanisms. However, the physiological and pathological status of the source cell and the type of source and recipient cells determine the mechanism used and its effect.

## Function of Exosomes in Living Organisms

### Protection against Bacterial and Viral Infection

**FIGURE 2:**
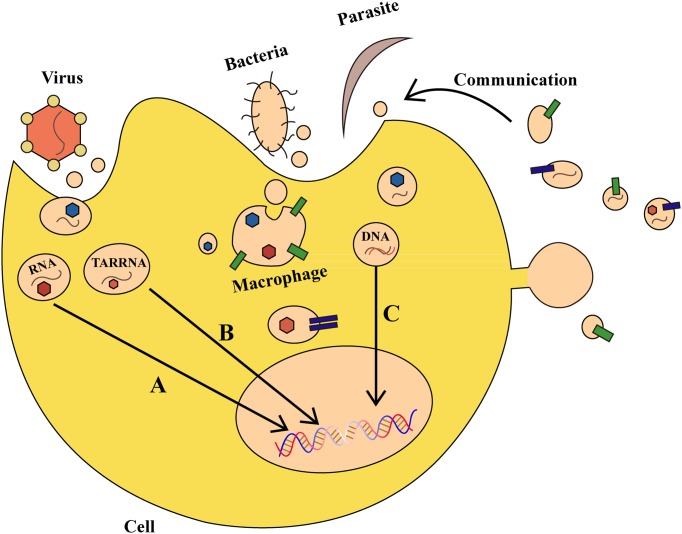
**The role of exosomes in bacterial, parasitic, or viral infections. (A)** Virus-derived exosomes contain proteins and RNAs that act on specific DNA regions and not only regulate gene expression in host cells, but also alter their survival condition. **(B)** Virus-secreted exosomes transfer trans-activating RNA (TAR-RNA) to stimulate expression of intracellular pro-inflammatory cytokines. **(C)** Bacteria and parasites can transfer DNA that encodes drug-resistance to other pathogens, thereby promoting the growth of pathogens. Moreover, an infected cell can use such communication as a defense mechanism.

There are over one hundred million people worldwide suffering from diseases caused by bacterial, parasitic, or viral infections, such as malaria and acquired immune deficiency syndrome (Murray et al., 2014). It was discovered that exosomes have a role in the pathogenesis of many bacterial, parasitic, or viral infectious diseases (**Figure 2**). Virus-infected cells secrete protein, lipids and RNA through exosomes, thereby enabling it to cause further infection in the host (Schwab et al., 2015). After infecting host cells, human immunodeficiency virus-1 uses exosomes to transfer trans-activating RNA (TAR-RNA), which stimulates the expression of intracellular pro-inflammatory cytokines (Sampey et al., 2016). Nakai et al. (2015) found that hepatocytes infected by the hepatitis C virus can secrete exosomes having RNA of the hepatitis C virus, which inactivates expression of TLR3, and mediates maturation of DCs. Additionally, the herpes virus takes advantage of the local microenvironment to release exosomes that have the virus and the Fast ligand, which after causes apoptosis of receptor cells through the extrinsic pathway.

Parasites can also secrete exosomes or other kinds of EVs, which can be internalized and absorbed by host cells. Marcilla et al. (2012) found that EVs secreted by the liver fluke and *Echinostoma* contain the same protein analogs as mammalian exosomes, which results in their internalization and absorption by intestinal epithelial cells in rats. Other studies demonstrated that erythrocytes infected by transgenic *Plasmodium falciparum* can transfer biological target DNA that encodes drug-resistance to other malaria parasites, thereby promoting pathogen growth (Regev-Rudzki et al., 2013). Exosomes secreted by *Leishmania* can enter neutrophils and be ingested by macrophages, which results in the selective release of interleukin-8 (IL-8) by macrophages. The exosomes released by *Leishmania* can also modulate immunity by promoting secretion of the immunosuppressive factor interleukin-10 (IL-10) or by conditionally inhibiting secretion of tumor necrosis factor (TNF) caused by interferon-γ (IFN-γ) (Ger et al., 2005; Silverman et al., 2010). Therefore on one hand, bacteria and parasites can communicate with host cells through exosomes and other vesicles, but on the other, host cells also use such communication as a defense mechanism. For example, during *Plasmodium* infection, a cell membrane can release microvesicles that induce an increase of CD40 on the surface of an antigen-presenting cell, which causes inflammation by stimulating T cells and other effects (Couper et al., 2010). Collectively, exosomes and other EVs can affect the outcome of parasite infection by regulating the interplay between parasite and host cells. Vesicles can be a defense-related role in antigen-presenting and host cells by transmitting signals between parasite and parasite, parasite and host, and host cells and the environment. The development of approaches to block the exosomes of viruses and parasites to curb virus infections or inflammatory reactions would be a major advancement in infection control that may confer benefits worldwide.

### Roles of Exosomes in Tumor Progression and Metastasis

**FIGURE 3:**
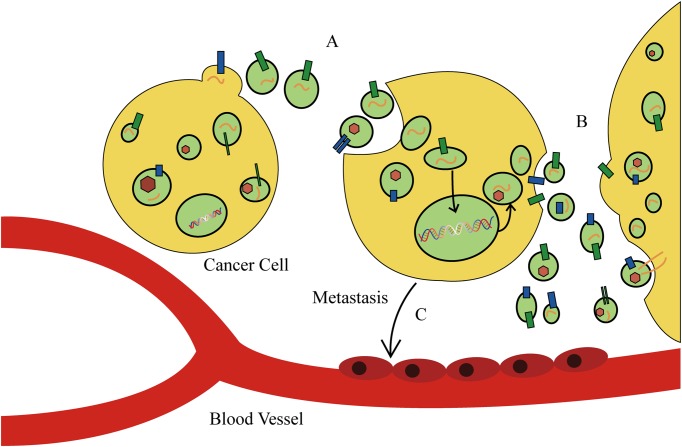
**The function of exosomes in cancer cell behavior and tumor microenvironment. (A)** Tumor-derived exosomes can promote tumor formation by regulating the synthesis of cell-independent microRNA (miRNA). **(B)** Cancer cells secrete exosomes to modulate the tumor microenvironment. **(C)** Tumor-derived exosomes direct metastatic organotropism of cancer cells.

The discovery of exosomes, and in particular, their role in mediating the transport or “traffic” of biological materials, has explained various pathological and physiological phenomenon that cannot be explained by intercellular message delivery. As a new model of mediating intercellular information exchange, exosomes transport oncogene materials and proteins in tumor initiation and progression (**Figure 3**). Recent studies have elaborated on the prominent role of exosomes in tumor carcinogenesis. Melo et al. (2014) reported that tumor-derived exosomes can promote tumor formation by regulating the synthesis of cell-independent miRNA. The miRNAs transferred by exosomes affect tumor initiation and progression in a Dicer-dependent manner. Using a triple SILAC-based quantitative proteomic analysis, Clark et al. (2015) found that oncoproteins, including *EGFR*, *GRB2*, and *SRC*, were enriched in exosomes derived from non-small cell lung cancer cells, which could actively promote the proliferation of recipient cells. Similarly, it was found that EGFRvIII and miR-21 levels in exosomes of patients with glioblastoma were upregulated, and that glioblastoma cells containing RNA- and angiogenesis-relevant proteins could induce tumor growth (Saadatpour et al., 2016). In addition, differences in exosomal contents were reported to distinguish cancer cells from normal cells in patients with other types of cancer, such as prostate cancer, gastric cancer, and laryngeal squamous cell carcinoma (Silva et al., 2015). These studies have identified enriched bioactive cargos in exosomes that can contribute to cancer progression, and therefore, may provide opportunities for the novel development of exosome-based biomarkers and therapies for cancer.

Tumor-derived exosomes can also alter the tumor microenvironment. Cui et al. (2015) found that exosomes derived from lung cancer cells can selectively transport miRNA-210 into endothelial cells and promote the formation of tumor blood vessels. He et al. (2015) and Mao et al. (2016) revealed that exosomes derived from metastatic liver cancer cells have many cancer-specific RNAs and proteins including the *MET* oncogene, members of the S100 protein family, and microencapsulated proteins. These microvesicles significantly enhance mobility and invasiveness of immortalized stem cells, which are initially immobile. Alečković and Kang (2015) and He et al. (2015) found that astrocyte-derived exosomes transport miRNA-19a, which reversibly decreases expression of phosphatase and tensin homolog (PTEN) in cancer cells, and promotes metastasis of tumor cells into the brain (Zhang et al., 2015). In addition, tumor-derived exosomes direct metastatic organotropism of cancer cells (Alečković and Kang, 2015). Exosome proteomic analysis revealed that integrin expression patterns of cancer cells contribute to metastatic tendency. For example, integrin α_6_β_4_ and integrin α_6_β_1_ are related to metastasis of tumor cells in lung, while integrin α_v_β_5_ is linked to liver metastasis. Depleting integrins α_6_β_4_ and α_v_β_5_ reduced exosome uptake and resulted in the inhibition of lung and liver metastasis, respectively. Therefore, integrins found on specific tumor-derived exosomes can be used to predict organ-specific cancer metastasis and are new targets for developing therapeutic strategies for cancer metastasis (Hoshino et al., 2015). However, tumor-secreted exosomes that alter tumor microenvironments and promote tumor progression can also exert the opposite effect. Exosomes released by colorectal cancer cells cause mesenchymal stromal cell dysfunction, which hinders tumor development. Although admittedly more complex than our current understanding, there is enormous potential for the development of novel exosome-based anti-tumor therapies for several types of cancer.

### Adjusting Tumor Immunity

**FIGURE 4:**
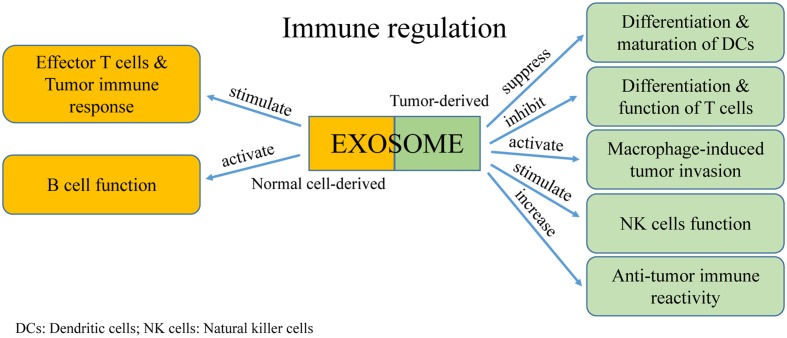
**The function of exosomes in immune regulation**.

Extracellular vesicles were first discovered as a mechanism for reticulocytes to transfer transferrin during maturation. Because they contain major histocompatibility complexes (MHCs) and present antigens, EVs rapidly attracted the attention of immunologists. An increasing number of studies reported the significant roles of EVs in regulating tumor immunity (**Figure 4**). Moreover, communication between immune cells and cancer cells via exosomes have dual effects in modulating tumor-related immunity because exosomes can mediate both immune activation and suppression, thereby affecting tumor development.

### Mediating Immune Suppression of Tumor Host Cells

#### Inhibiting Differentiation and Maturation of DCs

Dendritic cells are specialized antigen-presenting cells that are formed from bone marrow stem cells. They operate by presenting antigens and initiate early T cell responses including anti-tumor reactions. In tumor microenvironments, exosomes from tumor cells can suppress the differentiation and maturation of DCs. This results in bone marrow precursor cells becoming immature bone marrow cells, which promotes tumor progression as marrow-derived suppressor cells (Condamine and Gabrilovich, 2011). Using a standard murine DC culture system *in vitro*, it was found that addition of tumor-derived exosomes induced expression of interleukin-6 (IL-6) and inhibited differentiation of bone marrow precursor cells into DCs (Liu et al., 2010). In addition, tumor-derived exosomes can activate differentiation of CD14^+^ monocytes into CD14^+^ HLA-DR-/low cells, which suppress the differentiation and cell lysis ability of T cells (Valenti et al., 2006). It is currently understood that the underlying mechanism is primarily related to proteins transported by the exosomes, such as transforming growth factor-β (TGF-β), IL-6, and prostaglandin E2 (PGE2) (Yu et al., 2007; Liu et al., 2010).

#### Activation of Macrophages in Tumor Invasion and Metastasis

Macrophages adjust their physiological activity depending on the environment, especially in a tumor microenvironment (Marton et al., 2012). Activation of macrophages by tumor-derived exosomes regulates tumor invasion and metastasis through reducing the release of tissue inhibitor of metalloproteinase-1 (TIMP1), IFNγ, and interleukin-16 (IL-16), and by increasing secretion of IL-8, C-C motif ligand 2 (CCL2), major intrinsic proteins (MIPs), and interleukin-1 receptor antagonist (IL-1Ra). Tumor-derived exosomes also cause increased Wnt5a expression in macrophages. However, this process is bidirectional because Wnt5a can transfer from macrophages into tumor cells via exosomes, which results in the downstream activation of the β-catenin-independent Wnt signaling pathway in target cells (Menck et al., 2013). This unanticipated cycle provides researchers with an intriguing mechanism in understanding macrophage-induced tumor invasion. Recently, a study found that exosomes released by pancreatic ductal adenocarcinoma promote the formation of pre-metastatic niche and increase the liver metastasis of pancreatic ductal adenocarcinoma (Giovannetti et al., 2017).

#### Regulating the Cytotoxicity of Natural Killer Cells

Natural killer (NK) cells are typical cytotoxic lymphocytes that are part of the innate immune system of the body, which mainly act as killer cells (Vivier et al., 2012). Exosomes derived from human hepatocarcinoma cells treated by different anti-cancer drugs (e.g., Paclitaxel, Cisplatin) are extremely abundant in heat shock proteins (HSPs), which can stimulate NK cells to generate anti-tumor responses (Lv et al., 2012). In contrast, breast cancer-derived exosomes directly repress NK cell cytotoxicity, and furthermore, cause immune suppression (Wen et al., 2016). These varying effects may be because tumor-derived exosomes can segregate macrophages from activating receptors, such as NKG2D, NKP30, NKP46, and NKG2C. Therefore, the cytotoxic effects of NK cells are prevented in a tumor microenvironment. Studies have found that prostate cancer-derived exosomes suppress cytotoxicity of NK cells by reducing the expression of NKG2D on the cell surface. This effect may be because tumor-derived exosomes elevate the level of TGF-β, which induces immunosuppression (Hedlund et al., 2011; Szczepanski et al., 2011).

#### Impairing Cytotoxic Lymphocyte Immune Response and Regulating T Cell Function

Effector CD4^+^ and CD8^+^ T cells are vital in the anti-tumor immune process. However, tumor-derived exosomes can affect the proliferation, activation, and apoptosis of these types of T cells. Exosomes derived from human nasopharyngeal carcinoma cells carry miRNAs that inhibit proliferation and differentiation of T cells into Th1 and Th17 cells, and promote the formation of regulatory T cells (Klibi et al., 2009). The overexpressed miRNAs found in these exosomes mainly consist of hsa-miR-24-3p, hsa-miR-891a, hsamiR-106a-5p, hsa-miR-20a-5p, and hsa-miR-1908. They reduce activity of the mitogen-activated protein kinase (MAPK) signaling pathway through downregulating the phosphorylation of extracellular regulated protein kinase (ERK), signal transducer and activator of transcription 1 (STAT1), and STAT3. In a rat glioblastoma tumor model, it was found that exosomes decreased activation of CD8^+^ T cells and reduced expression of IFN-γ and granzyme B (Liu et al., 2013). Cancer cell-derived exosomes contain TNF-α, which affects the interaction of the TCR-CD3 complex, thereby blocking the activating signals of CD4^+^ and CD8^+^ T cells (Söderberg et al., 2007). Furthermore, Fas ligand-positive exosomes derived from cancer cells from a patient with oral cancer results in apoptosis of activated T cells (Taylor and Gerçel-Taylor, 2005), a finding which suggests exosomes have a role in T cell apoptosis.

#### Regulating B Cell Function

Exosomes secreted by activated B cells can stimulate effector T cells to attack tumor cells and cause a tumor immune response. However, the role of tumor-derived exosomes in modulating regulatory B cells is unclear. Exosomes that are secreted by cardiac endothelial cells contain integrin αvβ6 and stimulate B cells to produce TGF-β, which inhibits T cell proliferation (Song et al., 2014). Similarly, exosomes derived from mycoplasma-infected tumor cells contain original mycoplasma components, which induce B cell generation and results in inhibition of T cell activation (Yang et al., 2012). These findings demonstrate how mycoplasma-infected tumor cells regulate the function of B cells, and moreover, demonstrates how pathogen-derived exosomes use B cells to disrupt initiation of the immune response and avoid detection by immune surveillance.

#### Mediating Activation of the Anti-tumor Immune Response

Exosomes can also stimulate the immune system to produce anti-tumor responses through cell–cell communication, of which the major component of this process is the antigen-presenting cell. It was shown that B cell-derived exosomes load many antigen-presenting molecules, such as MHC I, MHC II, and CD86. MHC II-presenting antigens can activate CD4^+^ T cells after follicular DC processing (Nazimek et al., 2015). Tumor cell-derived exosomes can increase anti-tumor immune reactivity by transferring tumor antigens to DCs, which ultimately present as a cytotoxic lymphocyte (Di et al., 2015). Mast cell-secreted exosomes contain functional proteins that indirectly activate B and T cells, and regulate specific immune responses (Mion et al., 1978), whereas DC-derived exosomes destroy tumors cells by stimulating the expression of MHC I and CD86 on CD8^+^ T cells (Sadallah et al., 2011). It was also found that exosomes contain different amounts of immune-related molecules, such as antigen-presenting MHC I and II, HSPs, and co-stimulatory molecules (Clayton et al., 2001). Furthermore, human NK cell-derived exosomes isolated from the blood of healthy donors expressing NK markers, such as CD56, perforin, and FasL, exhibited cytotoxic effects against several types of cancer especially those of hematological origin as well as moderated immune activities both *in vitro* and *in vivo* (Lugini et al., 2012). These findings demonstrate that in addition to having a role in signal transmission, exosomes have definite immune functions as well. Therefore, exosomes are frequently being considered for use as non-cellular vaccines and have been applied in tumor cell immunotherapy.

Exosomes can have tumor-associated antigens, which can attract antigen presenting cells. Tumor antigens on exosomes can be transported into DCs and stimulate specific cytotoxic lymphocyte responses (Kahlert and Kalluri, 2013). At present, the tumor-associated antigens discovered in exosomes consist of HSP70-80, Her2/Neu, Mart1, TRP, and gp100 from melanoma tumor cells, and intracisternal particle protein A and HSP70 from plasmacytoma cells. However, these exosomes result in weak anti-tumor immune responses, and are not difficult to induce immune tolerance.

## Current Research Progress

As investigation in exosomes increased and our understanding deepened, a distinctive pattern emerged in the field of exosome research in which current efforts focused on inter-cell communication mediating cell behavior, biomarker screening, or drug delivery. Acquired drug resistance in tumors and disease recurrence remains a significant challenge in oncotherapy. Qu et al. (2016) found that exosomes secreted from renal cell carcinoma cells with sunitinib resistance contain a specific long non-coding RNA that confers sunitinib resistance via competitively binding miR-34/miR-449 to facilitate AXL and c-MET expression in renal cell carcinoma cells. In addition, RNA from primary tumor exosome induced TLR3 pathway activation in parent lung epithelial cells, which may promote metastatic niche formation and provide underlying targets to impede tumor metastasis in lung (Liu et al., 2016). There is a body of evidence that exosomes have a role in the immune process. One study found that exosomes transferred lipopolysaccharides to host cells during Gram-negative bacteria infection, which activates caspase-11 (Vanaja et al., 2016). Further illustration of the importance of exosomes in the inflammation response was demonstrated by Han et al. (2016) who reported that an interaction between microvesicles and insulin-like growth factor 1 (IGF-1)-dependent macrophages and epithelial cells affected tissue inflammation. Considering another major focus of exosome research, a drug carrier materials study by Votteler et al. (2016) reported the design of a biological structure called “enveloped protein nanocages” that can be released like viruses from human cell microvesicles. These synthesized structures have membrane-binding and self-assembly properties, and can recruit endosomal sorting complexes required for transport. As examples of the other major research focus of exosomes, miRNAs in salivary exosomes are considered potential biomarkers of aging (Machida et al., 2015), while exosomes from a tumor microenvironment are prominently associated with cancer cell metabolism (Zhao et al., 2016). Exosomes containing miR-24-3p depress T cell function and may serve as a prognostic marker for nasopharyngeal carcinoma (Ye et al., 2016). In addition, a recent study found that bovine milk-derived exosomes can be used for oral delivery of the chemotherapeutic drug paclitaxel, which elevates drug stability and reduces systemic and immunogenic toxicities compared to administering the drug alone (Agrawal et al., 2017).

### Progress in Clinical Research

As a communication messenger between cells, the potential role of exosomes in the clinical treatment and prevention of diseases has gradually emerged. For example, the levels of exosomes found in plasma from patients with different types of tumors are markedly higher than those found in plasma from healthy individuals, a finding which may be clinically used toward the identification of tumor progression and avoidance of immune surveillance (Logozzi et al., 2009; Raposo and Stoorvogel, 2013).

#### Tumors

Various tumor-derived exosomes have been identified that harbor several specific molecules from different types of tumors in patients with cancer, which signifies their putative importance as novel biomarkers for early diagnosis or targeted tumor treatment (Fais et al., 2016). Exosomes can be obtained from many types of body fluids namely, plasma, serum, urine, peritoneal lavage fluid, and gastric juice. Isolated exosomes from biological samples of patients with tumors contain tumor-specific molecules that can be used as novel biomarkers for the early diagnosis of cancer, in personalized medicine for tumor therapy, and in evaluation indexes for prognosis (Zocco et al., 2014).

Prostate-specific antigen is detected in exosomes from the plasma and urine of patients with prostate cancer. Prostate-specific antigen is currently used to screen patients with prostate cancer and for early diagnosis. However, in combination with other exosome-derived molecules, such as TMPRSS2:ERG2, TM256, ADIRF, LAMTOR1, and PCA3 mRNA, methods can be developed that may minimize or even avoid the risk of a false positive diagnosis of prostate cancer (Mitchell et al., 2009; Mizutani et al., 2014; Cheng, 2015; Tosoian et al., 2016). In addition, survivin and AGR2 splice variants may also be good indicators of prostate cancer as biomarkers of early diagnosis (Neeb et al., 2014; Lin et al., 2015). For other types of cancer, the number of exosomes expressing CD63^+^ and/or caveolin-1^+^ are significantly increased in the plasma of patients with melanoma compared to that in healthy individuals, a finding that exhibits a higher detection sensitivity than that of conventional biomarkers. CD63^+^ and caveolin-1^+^ are mutually related, and in conjunction with other biomarkers, such as p-Met, TYRP2, HSP70, HSC70, and VLA-4, this association increases the predictive accuracy of early diagnosis and prognosis of melanoma (Logozzi et al., 2009; Peinado et al., 2012). Recently, a study of patients with breast cancer detected glypican-1- and CD24-positive exosomes in patient serum samples, which may be used as biomarkers of breast cancer. Moreover, glypican-1 may be used with KRAS in the diagnosis of pancreatic cancer (Jia et al., 2017). For patients with gastric cancer, putative biomarkers from exosomes obtained from plasma, peritoneal lavage fluid, and gastric juice are CCR6, HER-2/neu, methylated LINE 1, and SOX1 as DNA-based markers as well as miR-21 and miR1225-5p as RNA-based markers (Baran et al., 2010; Tokuhisa et al., 2015; Yamamoto H. et al., 2016). In addition, exosomes from patients with bladder cancer express elevated levels of EDIL-3/Del1, TACSTD2, LASS2, and GALNT1 with no expression of ARHGEF39 and FOXO3 (Chen et al., 2012; Beckham et al., 2014; Perez et al., 2014).

As described above and to our knowledge, exosomes from different biological sources play various roles in different physiological and pathological processes in the human body. Based on function, exosomes especially derived from immune cells may be developed into nanomedicines or vaccines for patients with tumors. It was reported that DC-derived exosomes harboring functional MHC/peptide complexes capable of inducing a T cell immune response were used to treat melanoma and non-small cell lung cancer, which have both entered into Phase I clinical trials (Escudier et al., 2005; Tacken et al., 2007). Furthermore, a Phase II clinical trial using exosomes derived from DCs as a modified vaccine is underway to determine whether it can regulate an anti-tumor immune response in patients with unresectable non-small cell lung cancer (Zitvogel et al., 1998). Similarly, somatic stem cell-derived exosomes, especially mesenchymal stem cell-derived, can exert significant effects on immune suppression and regenerative therapies. Mesenchymal stem cell-derived exosomes have been tested as vaccines in a Phase I clinical trial for treatment of graft-versus-host disease in patients with type I diabetes (Kordelas et al., 2014).

As acidic vesicles, tumor exosomes as drug-delivery vehicles can effectively ensure drugs maintain their native form and promote drug uptake by tumor cells, which circumvents the intrinsic resistance of cells to accumulate cytotoxic drugs because of the low pH of the tumor microenvironment (Federici et al., 2014). An added advantage is that exosomes derived from normal human cells can also be used as a drug delivery system. Exosomes separated and purified from the culture supernatant of macrophages isolated from peripheral blood of healthy donors can transport acridine orange into human melanoma cells. Acridine orange destroys tumor cells following excitation with a light source at 466 nm (Iessi et al., 2017). Furthermore, exosomes secreted by M1-polarized, pro-inflammatory macrophages may be used as an immunostimulant with a cancer vaccine (Cheng et al., 2017). Both exosomes and miRNAs have a role in multi-drug resistance in tumors, which is believed to occur though regulating the expression of P-glycoprotein. P-glycoprotein transports drugs out of the cell membrane and attenuates the accumulation of anti-neoplastic drugs in tumor cells. miRNA-containing exosomes that target *ABCB1*, which encodes P-glycoprotein, may interfere with this process preventing the development of drug resistance in tumors (Bach et al., 2017).

#### Other Diseases

Since exosomes are considered significant moderators between bacteria and other infectious pathogens, they may be exploited as novel tools to diagnose and treat infectious diseases. Exosomes are secreted by infectious pathogens including bacteria, viruses, and fungi, and contain pathogen-associated molecular patterns that can be used to stimulate an immune response (Lener et al., 2015). Similar to the novel tumor treatment strategy, exosomes from immune cells especially DCs that are modified with a pathogenic microorganism may serve as anti-infectious agents. DC-derived exosomes pulsed with antigen from *Toxoplasma gondii* resulted in a systemic Th1-biased specific immune response, which conferred preventive effects against *T. gondii* infection in mice (Aline et al., 2004; Beauvillain et al., 2007). Furthermore, exosomes isolated from lethal *P. yoelii*-infected reticulocytes participate in immune regulation causing a noticeable decrease in the course of parasitemia (Martin-Jaular et al., 2011). One study reported that sand flies infected with *L. major* and *Leishmania* exosomes are co-ingested with the parasite during the insect’s bite *in vivo*, which affected the infectious process of the host and worsened the disease condition by over-inducing inflammatory factors (Atayde et al., 2015). Currently, a study found that insertion of HPV-E6 in engineered exosomes induced CD8^+^ T cell immunity against a tumor-associated antigen, a finding which demonstrated a putative anti-tumor therapeutic approach in a preclinical model (Manfredi et al., 2016). In addition, exosomes are associated with neurodegenerative diseases, such as Alzheimer disease (AD) and Parkinson disease. A recent study found that AD caused by the accumulation of β-amyloid (Aβ) peptides in senile plaques is related to an exosome-associated protein called ALIX, which suggests a significant role of exosomes in the pathogenesis of AD (Rajendran et al., 2006). Exosomes can influence Aβ accumulation and synaptotoxicity, and Aβ peptides (Aβ1-Aβ42) and tau proteins (total tau and phosphorylated tau) may be cerebrospinal fluid biomarkers for the diagnosis of AD degeneration (Joshi et al., 2015).

### Extraction and Isolation Technology for EV-Based Diagnosis

Currently, isolation methods of tumor-derived EVs are based on their size, density, and solubility, factors which are non-specific. Ultracentrifugation and filtration using a 0.22 μm-filter followed by ultracentrifugation and separation using a sucrose gradient can be used to obtain total exosomes, while ExoQuick precipitation can be used to isolate total EVs. For EV-based diagnosis, enzyme-linked immunosorbent assay-based approaches are considered the gold standard for single-plexed detection of low-abundancy proteins (ng/mL levels) with high sample throughput. For multiplexed detection, mass spectrometry is used for proteomic analysis of cancer-derived EVs. Because of the extreme complexity and considerable concentration ranges of biomolecules found in plasma samples, current analysis techniques face a great challenge. Numerous conventional methods, such as mass spectrometry, microarrays, and real-time PCR, have significant technical limitations in separation peak capacity, sensitivity, and dynamic range (Zocco et al., 2014).

## Discussion

Extracellular vesicles, including exosomes and other microvesicles, are the basic biological membrane-enveloped structure of organisms released by both normal cells and pathogens. They mediate communication between cells and participate in many physiological and pathological processes. The clinical potential of exosomes has been gradually realized as the breadth and scope of the different functions of exosomes have been determined. Exosomes transport several types of cellular materials, including DNA, mRNA, miRNAs, and proteins, and internalize many receptors, a process that promotes the activation of certain signaling pathways. Furthermore, exosomes can modulate cellular biological processes by changing the expression of biological (possibly therapeutic) targets on the surface of cells as well as regulate the expression of intracellular components. By synthesizing and releasing exosomes, tumor cells promote angiogenesis, which provides a microenvironment that favors proliferation and directional metastasis of tumor cells. Therefore, by targeting the bioactive molecules and surface antigens of exosomes, we can determine tumor progression at the initial stage and prevent metastasis in advance. In addition, tumor-derived exosomes mediate communication between tumor and immune cells, and induce immune activation or suppression in cells of the immune system. Thus, because exosomes regulate tumor immunity in a bidirectional manner the outcome of which is determined as a balance between opposing “yin and yang” effects, exosomes have a key role in anti-tumor immunity, which is of untapped clinical potential. Furthermore, exosomes obtained from viruses and parasites can promote pathogen infection, an understanding of which will enable the development of exosome-mediated strategies to counter infection of pathogenic microorganisms that affect millions of people worldwide.

Since the first meeting of the International Society for Extracellular Vesicles (ISEV) in 2013, researchers have invented many terms and names for secreted vesicles based on their different physiological functions including calcifying matrix vesicles that are involved in bone formation, tolerosomes that cause immune tolerance, and prostasomes, which are derived from prostate epithelium. However, “exosomes” and “microvesicles” have been widely applied and are more generic terms. To unify vesicle nomenclature, ISEV recommends using the term “extracellular vesicles” as a generic term for all secreted vesicles (Gould and Raposo, 2013). EVs include nanometer-scale vesicles (exosomes) as well as larger-scale nanometer vesicles (microvesicles). It has also been determined that only exosomes and larger vesicles have potential applications in disease diagnosis and nanomedicine development because these structures are relatively stable and have broad research prospects.

## Conclusion

Exosomes are a hot topic for targeted drug research. The design of non-drug small molecule pharmaceuticals that target the secretory pathways of exosomes and other microvesicles have enormous potential and significant prospects. In addition to using exosomes as a novel drug delivery vehicle, considerable efforts have been performed to develop exosome-derived drugs by modifying the contents or surface proteins of tumor-derived exosomes. However, both target specificity and clinical effectiveness require further improvements. Exosomes were originally considered unremarkable vesicle structures, but with our current understanding they have become a global research interest. Exosomes exhibit significant research prospects and exosomes and exosome-derived drugs have the potential to provide more effective methods in the treatment of clinical diseases as well as pathogenic infections. Furthermore, we strongly believe that exosomes will be a critical tool in the prognosis, diagnosis, and treatment of various diseases such as cancer and neurodegenerative disorders as well as bacterial, viral, and parasitic infections.

## Author Contributions

XS, PC, XC, YY, and JX contributed to the discussion and revisions to the manuscript. JW wrote the manuscript and JZ prepared the figures.

## Conflict of Interest Statement

The authors declare that the research was conducted in the absence of any commercial or financial relationships that could be construed as a potential conflict of interest.
